# Supplementary material for: Pt nanoshells with a high NIR-II photothermal conversion efficiency mediates multimodal neuromodulation against ventricular arrhythmias
Source: Nat Commun. 2024 Jul 28;15:6362. doi: 10.1038/s41467-024-50557-w (PMC11284209; doi:10.1038/s41467-024-50557-w)
Supplement: Supplementary file 1 — Supplementary Information [file 41467_2024_50557_MOESM1_ESM.pdf]

Supplementary Information

Pt nanoshells with a high NIR-II photothermal conversion efficiency mediates multimodal neuromodulation against ventricular arrhythmias

Chenlu Wang[1,†], Liping Zhou[2,3,4,5,6,7,8,†], Chengzhe Liu[2,3,4,5,6,7,8,†], Jiaming Qiao[2,3,4,5,6,7,8], Xinrui Han[2,3,4,5,6,7,8], Luyang Wang[1], Yaxi Liu[1], Bi Xu[1], Qinfang Qiu[2,3,4,5,6,7,8], Zizhuo Zhang[2,3,4,5,6,7,8], Jiale Wang[2,3,4,5,6,7,8], Xiaoya Zhou[2,3,4,5,6,7,8*], Mengqi Zeng[1], Lilei Yu[2,3,4,5,6,7,8*], Lei Fu[1,7,8,9*]

[1]College of Chemistry and Molecular Sciences, Wuhan University, Wuhan 430072, China. [2]Department of Cardiology, Renmin Hospital of Wuhan University, Wuhan 430060, China; [3]Hubei Key Laboratory of Autonomic Nervous System Modulation, Wuhan 430060, China; [4]Cardiac Autonomic Nervous System Research Center of Wuhan University, Wuhan 430060, China; [5]Hubei Key Laboratory of Cardiology, Wuhan 430060, China; [6]Cardiovascular Research Institute, Wuhan University, Wuhan 430060, China. [7]Taikang Center for Life and Medical Sciences, Wuhan University, Wuhan 430060, China. [8]Institute of Molecular Medicine, Renmin Hospital of Wuhan University, Wuhan 430060, China. [9]The Institute for Advanced Studies, Wuhan University, Wuhan 430072, China.

*E-mail: whuzhouxiaoya@whu.edu.cn; lileiyu@whu.edu.cn; leifu@whu.edu.cn

[†]These authors contributed equally to this work.

Contents

Supplementary Fig. 1 | Characterization of intermediate products and element mapping of PtNP-shell.

Supplementary Fig. 2 | SEM image of PtNP-shell.

Supplementary Fig. 3 | Characterization of PtNP-shell surface.

Supplementary Fig. 4 | XRD spectrum of PtNP-shell prior to reacting with KOH.

Supplementary Fig. 5 | The XPS survey spectra

Supplementary Fig. 6 | High-resolution XPS spectra and fitting results

Supplementary Fig. 7 | Hydrodynamic size of PtNP-shell@PEG.

Supplementary Fig. 8 | Hydrodynamic size of PtNP-shell

Supplementary Fig. 9 | Absorption of PtNP-shell.

Supplementary Fig. 10 | Measurement of PtNP-shell blackness.

Supplementary Fig. 11 | Temperature increase images of PtNP-shell of different concentrations at different time points.

Supplementary Fig. 12 | Temperature elevation curves of PtNP-shell with various power densities of NIR-II laser.

Supplementary Fig. 13 | The impact of PEG on the photothermal properties of PtNP-shell.

Supplementary Fig. 14 | Photothermal stability.

Supplementary Fig. 15 | PtNP-shell co-cultured with neurons.

Supplementary Fig. 16 | Western blotting for TRPV1 and TREK1 from canine NG and LSG tissues.

Supplementary Fig. 17 | Effect of PtNP-shell photothermal stimulation of NG.

Supplementary Fig. 18 | Visual graphical recording of NG neural activity after light activation.

Supplementary Fig. 19 | Direct effect of NIR irradiation of NG.

Supplementary Fig. 20 | Effects of PtNP-shell photothermal activation of NG on ventricular electrophysiology.

Supplementary Fig. 21 | Statistical analysis for the density of cells after photothermal activation of NG tissues in different groups.

Supplementary Fig. 22 | Effect of photothermal activation of NG on heart rate variability.

Supplementary Fig. 23 | Effect of PtNP-shell photothermal inhibition of LSG.

Supplementary Fig. 24 | Visual graphical recording of LSG neural activity after light inhibition.

Supplementary Fig. 25 | Direct effect of NIR irradiation of LSG.

Supplementary Fig. 26 | Effects of PtNP-shell photothermal inhibition of LSG on ventricular electrophysiology.

Supplementary Fig. 27 | Statistical analysis for the density of cells after photothermal inhibition of LSG tissues in different groups.

Supplementary Fig. 28 | Statistical analysis of recorded ventricular arrhythmia events post myocardial ischemia.

Supplementary Fig. 29 | Effect of photothermal inhibition of LSG on heart rate variability.

Supplementary Fig. 30 | Ganglion biocompatibility of targeted injections of PtNP or PBS after NIR-II irradiation and after 30 days of follow-up.

Supplementary Fig. 31 | Long term biosafety of PtNP-shell microinjection.

Supplementary Table 1 | ξ potential of bare PtNP-shell, PtNP-shell + KOH and PtNP-shell@PEG.

Supplementary Table 2 | Comparison of photothermal conversion efficiency.

Supplementary references

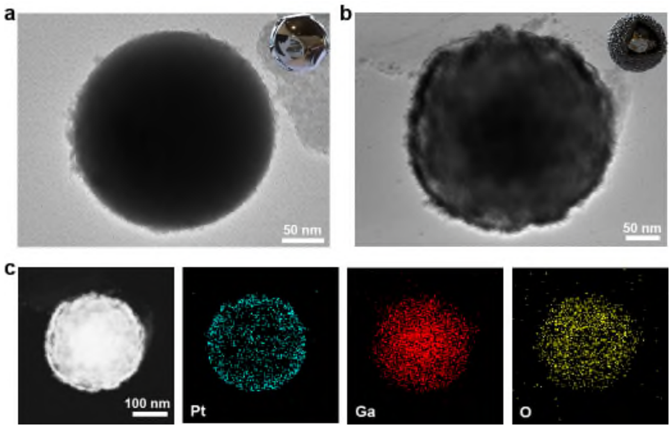

Supplementary Fig. 1 | Characterization of intermediate products and element mapping of PtNP-shell. TEM images of **a**, GaNPs (Inset: schematic diagram of GaNPs) and **b**, Ga@Pt NPs (Inset: schematic diagram of Ga@Pt NPs). **c**, element mapping of Ga@Pt NPs.

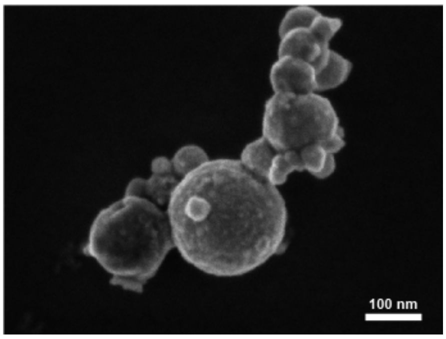

Supplementary Fig. 2 | SEM image of PtNP-shell.

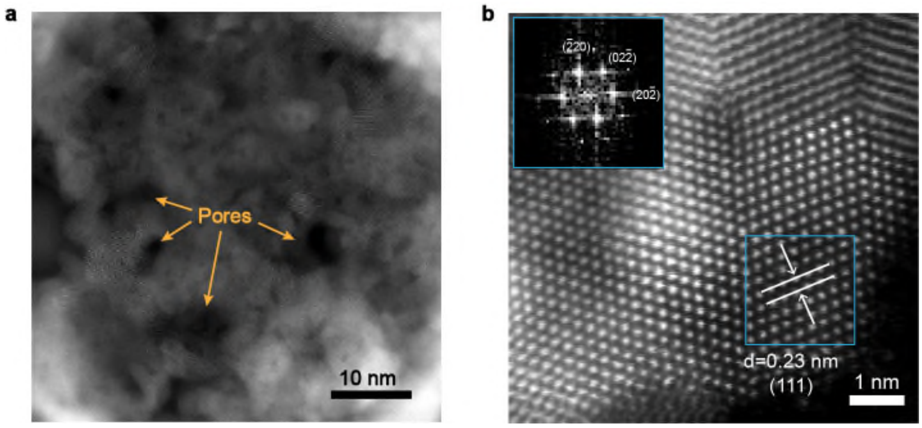

Supplementary Fig. 3 | Characterization of PtNP-shell surface. a, STEM images of PtNP-shell surface. **b**, HRTEM of PtNPs, the inset is the corresponding FFT pattern of the frame selection part.

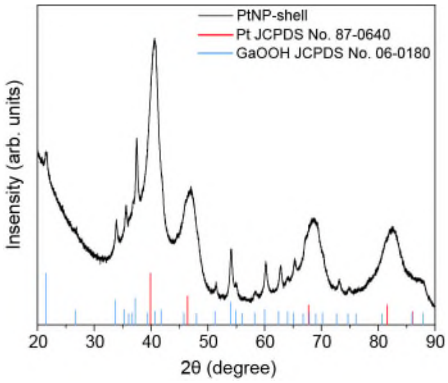

Supplementary Fig. 4 | XRD spectrum of PtNP-shell prior to reacting with KOH. Source data are provided as a Source Data file.

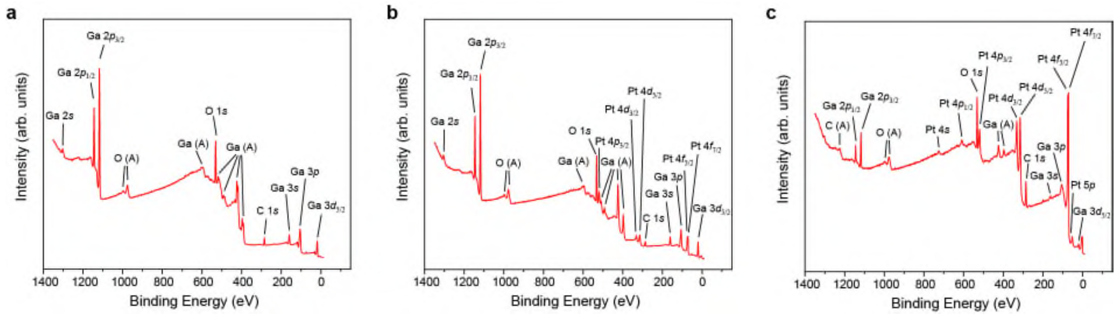

Supplementary Fig. 5 | The XPS survey spectra of **a**, GaNPs, **b**, Ga@Pt NPs and **c**, PtNP-shell. Source data are provided as a Source Data file.

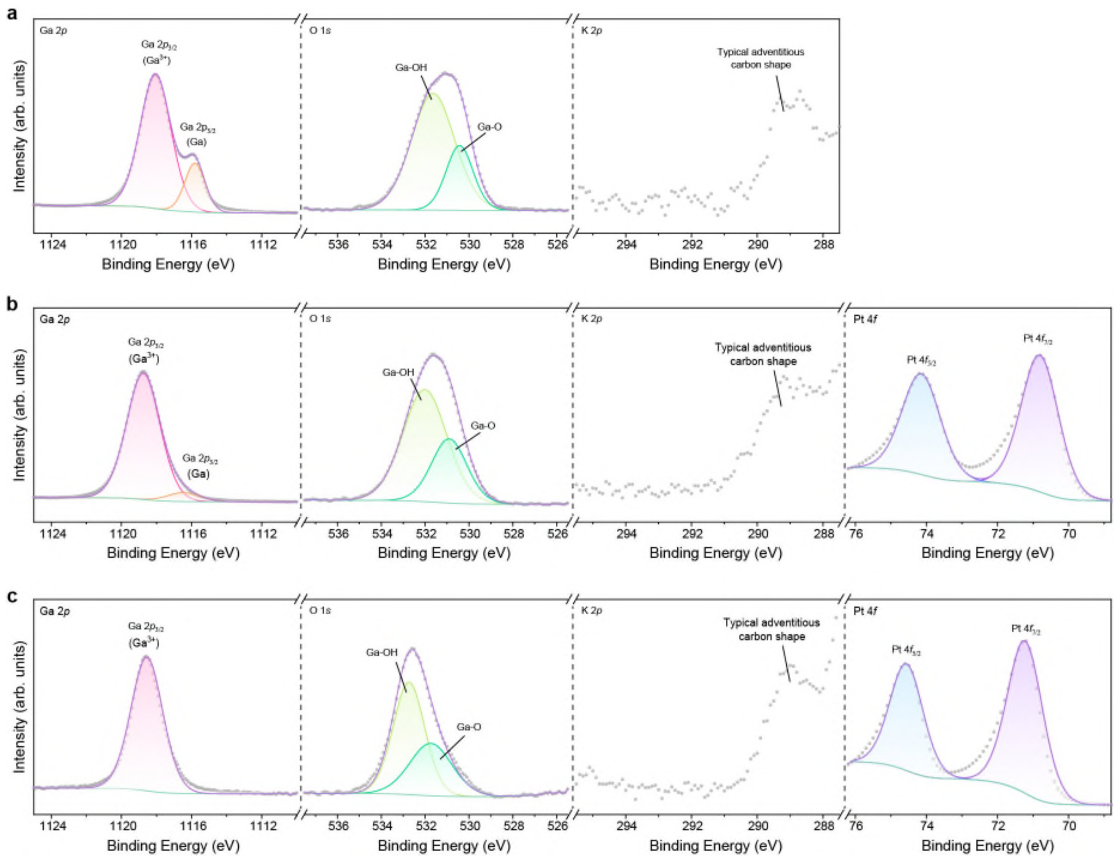

Supplementary Fig. 6 | High-resolution XPS spectra and fitting results of **a**, GaNPs, **b**, Ga@Pt NPs and **c**, PtNP-shell. Source data are provided as a Source Data file.

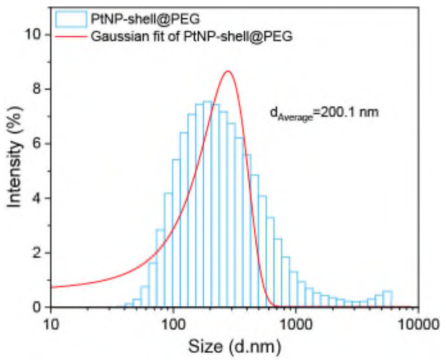

Supplementary Fig. 7 | Hydrodynamic size of PtNP-shell@PEG. Source data are provided as a Source Data file.

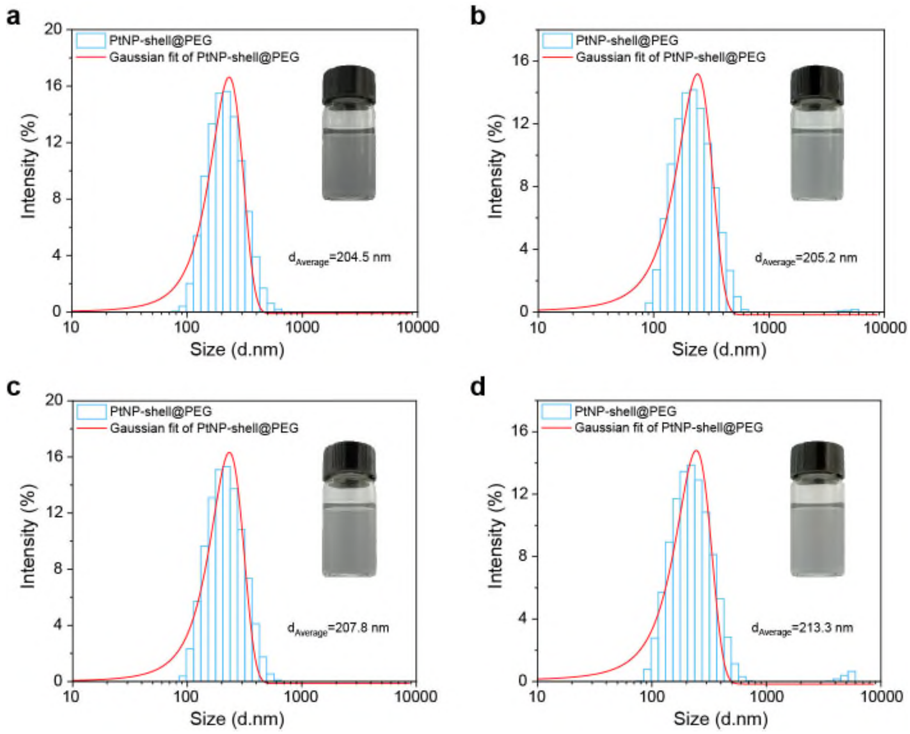

Supplementary Fig. 8 | Hydrodynamic size of PtNP-shell after a, 1, b, 4, c, 7, and d, 14 days of standing (Inset: digital photograph). Source data are provided as a Source Data file.

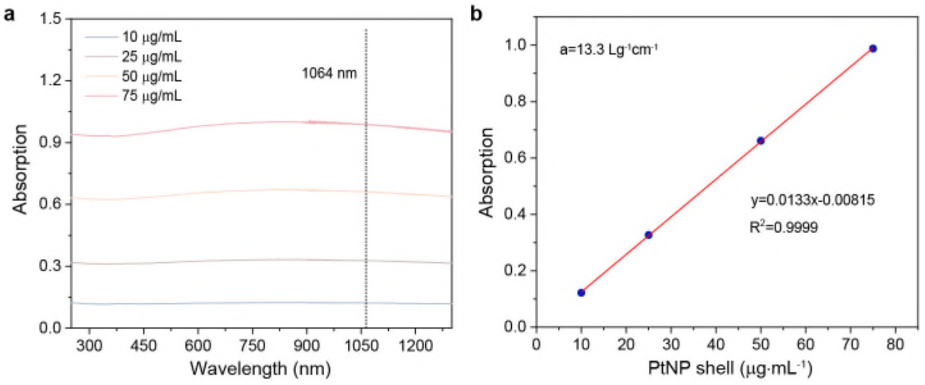

Supplementary Fig. 9 | Absorption of PtNP-shell. a, Absorption curves of PtNP-shell with different concentrations (10, 25, 50 and 75 μg·mL^{-1}). **b**, The Mass extinction coefficient of PtNP-shell at 1064 nm. Normalized absorbance intensity at λ = 1064 nm divided by the characteristic length of the cell (A/L) at different concentrations (10, 25, 50 and 75 μg·mL^{-1}). Source data are provided as a Source Data file.

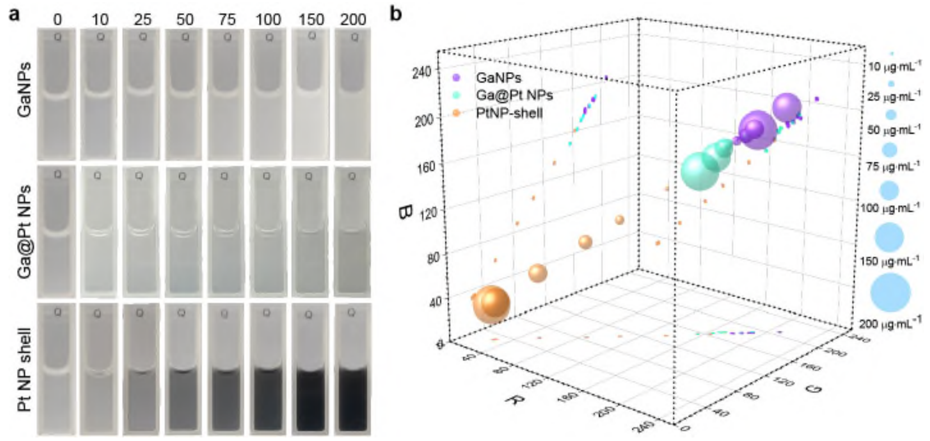

Supplementary Fig. 10 | Measurement of PtNP-shell blackness. a, Visual appearance of GaNPs, Ga@Pt NPs and PtNP-shell at different concentrations. **b**, Position of each color in the RGB cube, obtained by extracting the relative components of red, green and blue from Supplementary Fig. 10a. Source data are provided as a Source Data file.

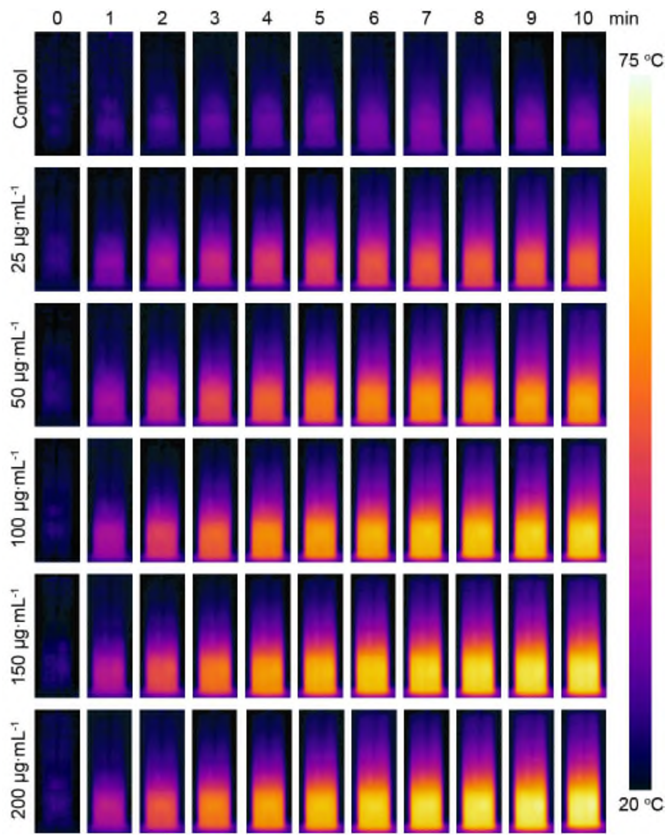

Supplementary Fig. 11 | Temperature increase images of PtNP-shell of different concentrations at different time points. Laser power density: 1 W·cm^{-2}.

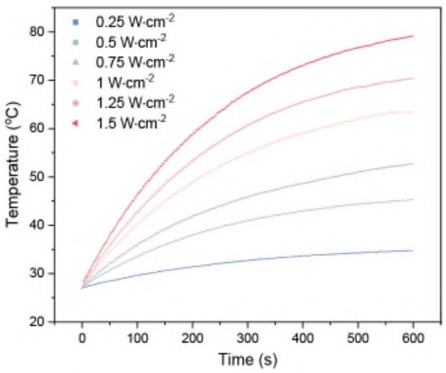

Supplementary Fig. 12 | Temperature elevation curves of PtNP-shell with various power densities of NIR-II laser. Source data are provided as a Source Data file.

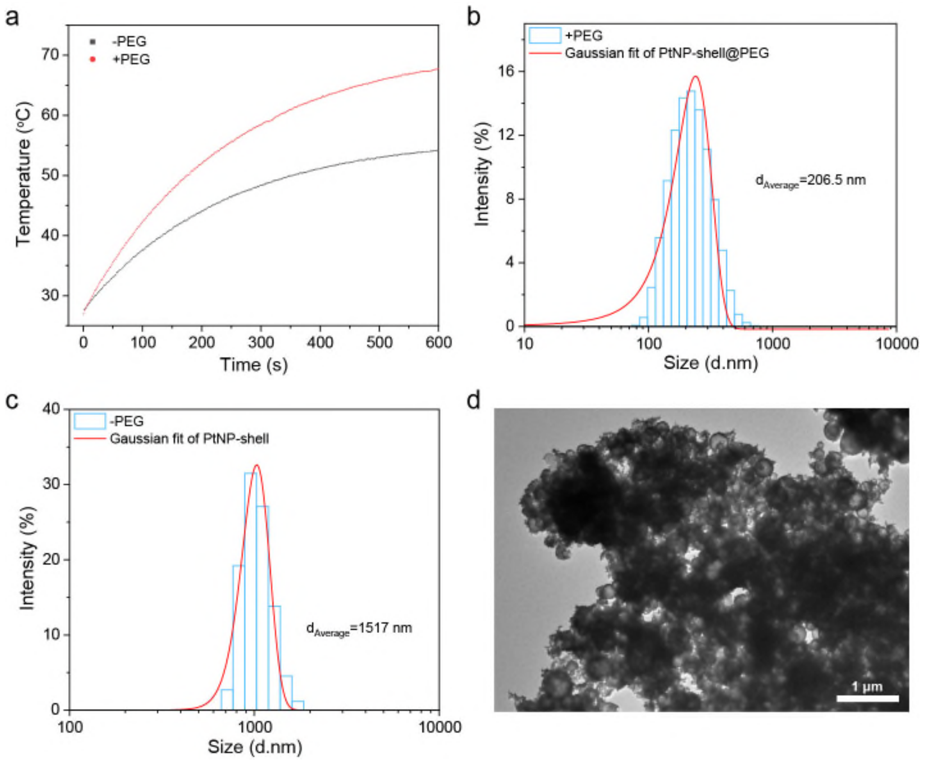

Supplementary Fig. 13 | The impact of PEG on the photothermal properties of PtNP-shell. **a**, Temperature elevation curves of SH-PEG modified and unmodified PtNP-shell. The hydrodynamic size of PtNP-shell **b**, before and **c**, after SH-PEG modification (after 600 s of 1064 nm laser irradiation). **d**, TEM image of PtNP-sehll before SH-PEG modification (after 600 s of 1064 nm laser irradiation). Source data are provided as a Source Data file.

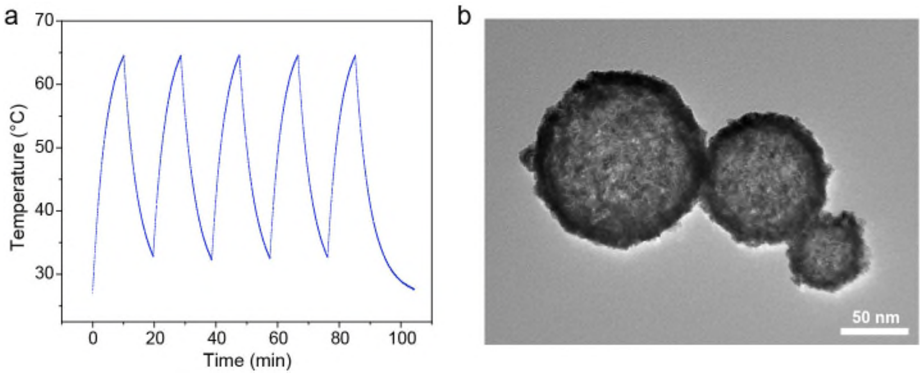

Supplementary Fig. 14 | Photothermal stability. a, Photothermal profiles of the suspension of PtNP-shell over five irradiation cycles. **b**, TEM image of PtNP-shell over five irradiation cycles. Source data are provided as a Source Data file.

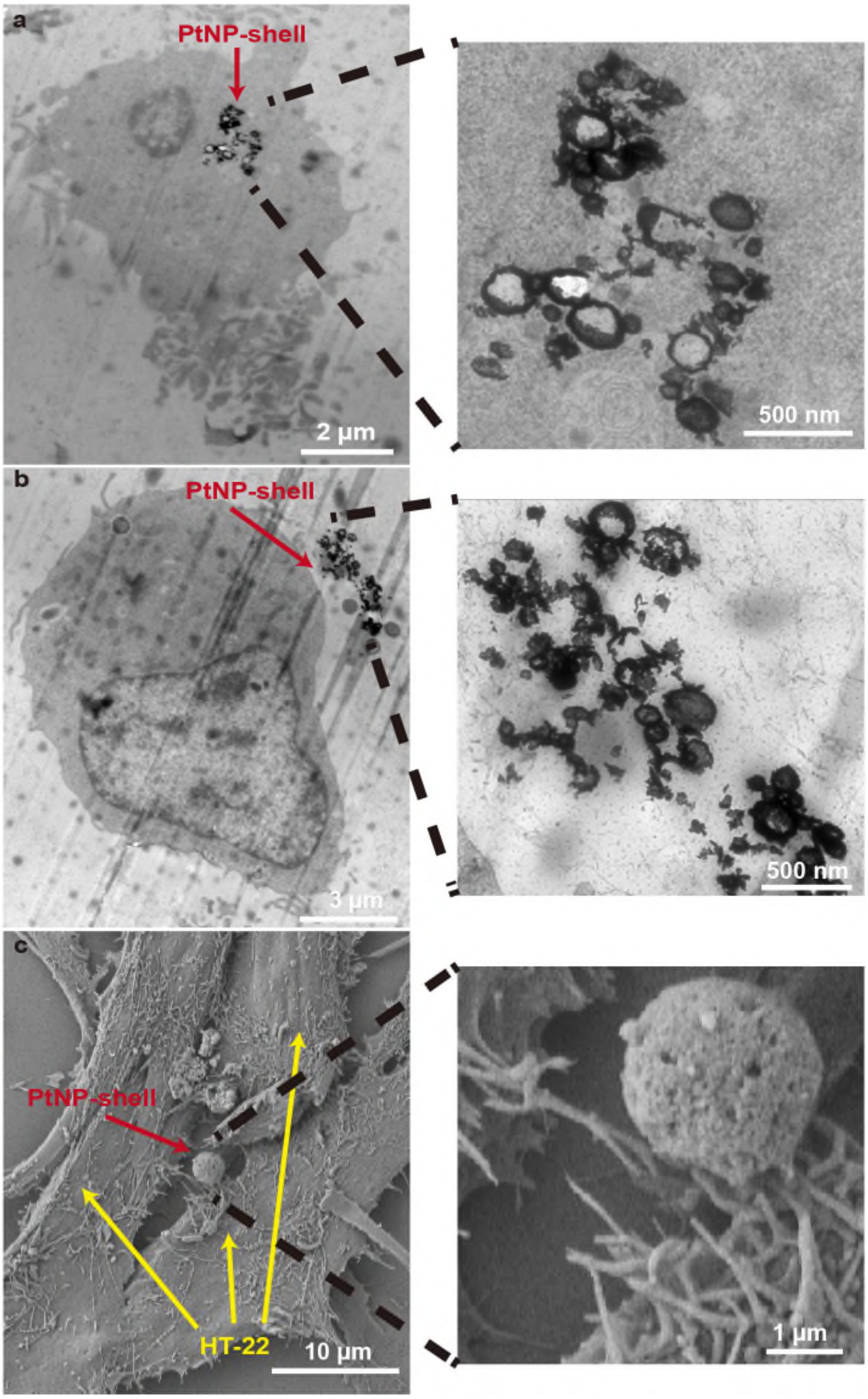

Supplementary Fig. 15 | PtNP-shell co-cultured with neurons. a and **b**, Cross-sectional TEM and **c**, SEM of the neurons incubated with PtNP-shell particles for 24 h.

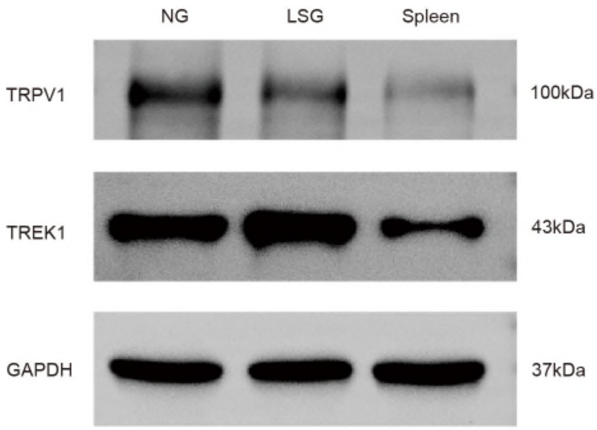

Supplementary Fig. 16 | Western blotting for TRPV1 and TREK1 from canine NG and LSG tissues. Representative western blotting bands for TRPV1, TREK1 from canine ganglion (NG and LSG) and spleen tissues, n = 4 biologically independent replicates. Source data are provided as a Source Data file.

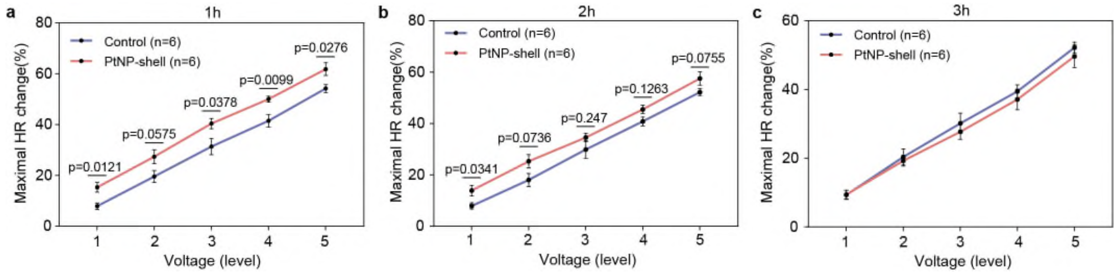

Supplementary Fig. 17 | Effect of PtNP-shell photothermal stimulation of NG. Maximal HR changes of beagles treatment with PtNP-shell or control from 1 to 3 hours after NIR irradiation, n = 6 biologically independent replicates. Data are shown as the mean ± S.E.M. Unpaired two-tailed Student's t-test was applied for statistical analysis of (a-c). Source data are provided as a Source Data file.

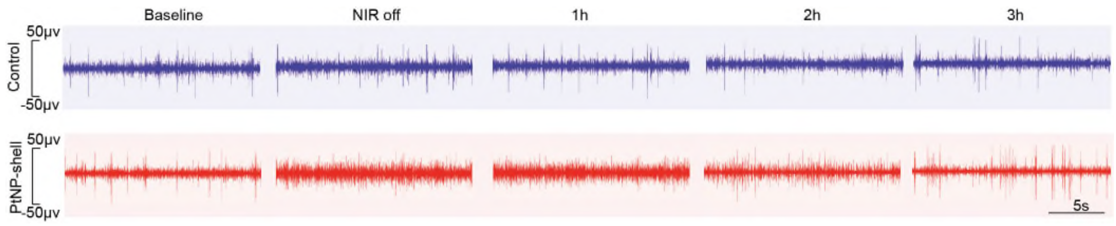

Supplementary Fig. 18 | Visual graphical recording of NG neural activity after light activation. Representative NG neural activity recordings of Beagles treatment with PtNP-shell or control at different time points.

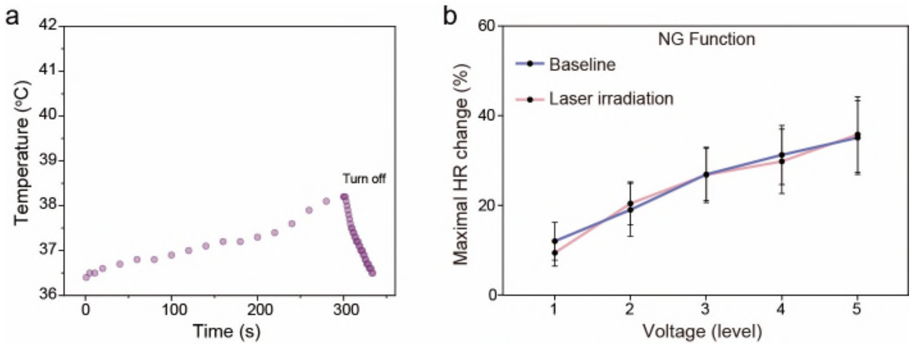

Supplementary Fig. 19 | Direct effect of NIR irradiation of NG. a, Local temperature curve of NG under NIR-II irradiation. **b**, Neural function of NG before and after NIR-II irradiation. Source data are provided as a Source Data file.

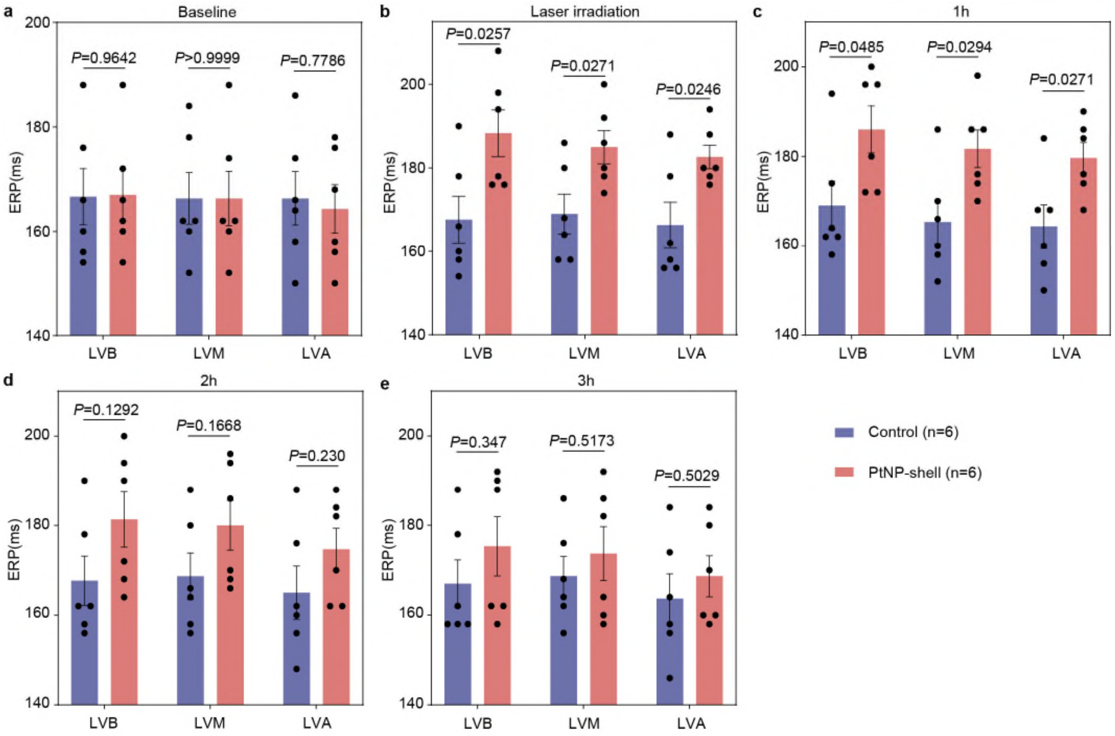

Supplementary Fig. 20 | Effects of PtNP-shell photothermal activation of NG on ventricular electrophysiology. Effects on ventricular ERP at different sites in beagles treatment with PtNP-shell or control at different time points, n = 6 biologically independent replicates. Data are shown as the mean ± S.E.M. Unpaired two-tailed Student's t-test was applied for statistical analysis of (a–e). Source data are provided as a Source Data file.

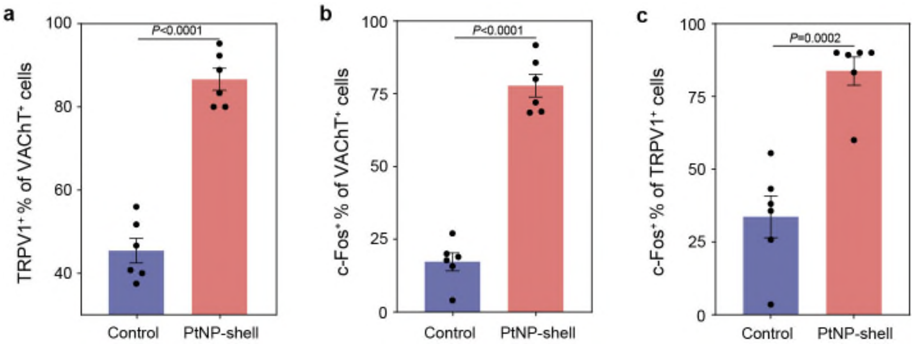

Supplementary Fig. 21 | Statistical analysis for the density of cells after photothermal activation of NG tissues in different groups. a, Percentage of TRPV1+ cells within the VAChT+ cell population. **b**, Percentage of c-Fos+ cells within the VAChT+ cell population. **c**, Percentage of c-Fos+ cells within the TRPV1+ cell population, n = 6 biologically independent replicates. Data are shown as the mean ± S.E.M. Unpaired two-tailed Student's t-test was applied for statistical analysis of (a-c). Source data are provided as a Source Data file.

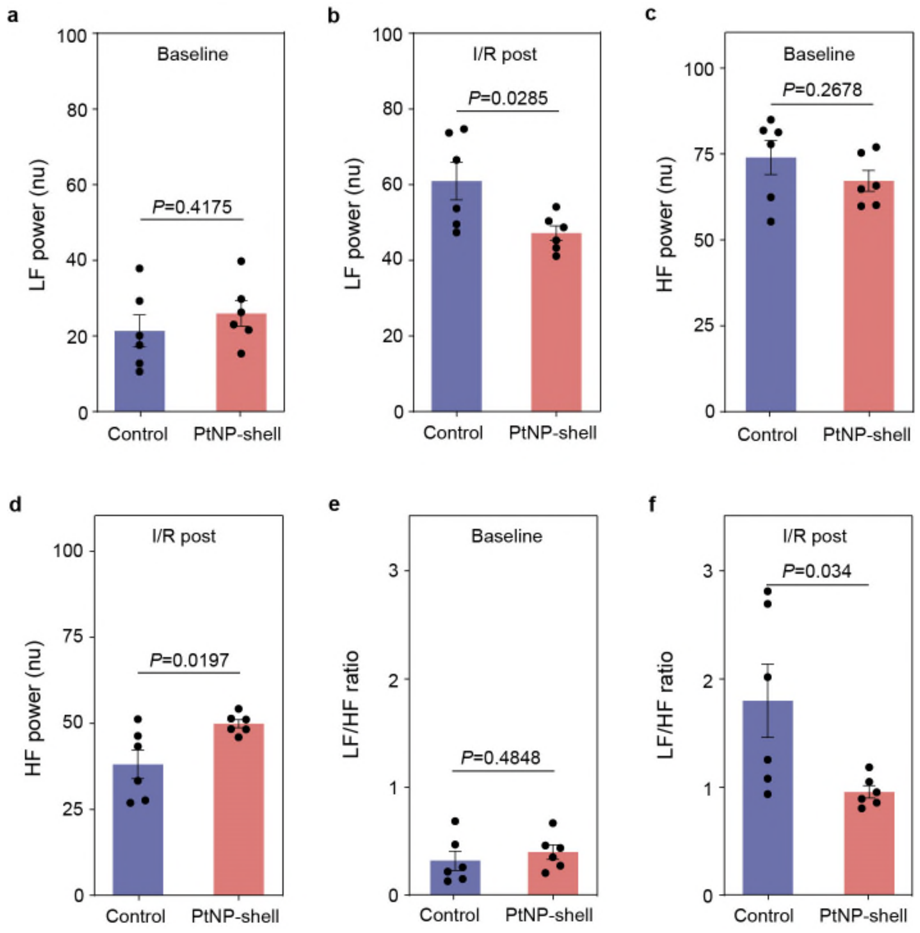

Supplementary Fig. 22 | Effect of photothermal activation of NG on heart rate variability. Comparison of LF between the two groups at **a**, baseline and **b**, post myocardial I/R injury. Comparison of HF between the two groups at **c**, baseline and **d**, post myocardial I/R injury. Comparison the ratio of LF/HF between the two groups at **e**, baseline and **f**, post myocardial I/R injury, n = 6 biologically independent replicates. Data are shown as the mean ± S.E.M. Unpaired two-tailed Student's t-test was applied for statistical analysis of (a–f). Source data are provided as a Source Data file.

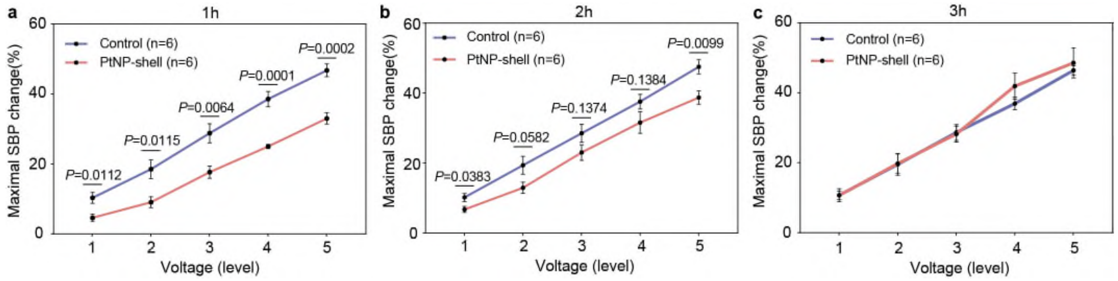

Supplementary Fig. 23 | Effect of PtNP-shell photothermal inhibition of LSG. Maximal SBP changes of beagles treatment with PtNP-shell or control from 1 to 3 hours after NIR irradiation, n = 6 biologically independent replicates. Data are shown as the mean ± S.E.M. Unpaired two-tailed Student's t-test was applied for statistical analysis of (a–c). Source data are provided as a Source Data file.

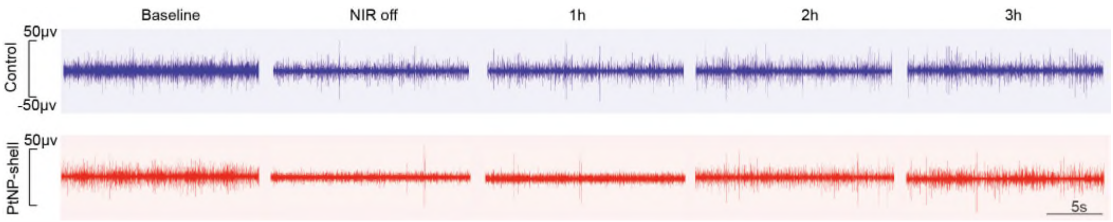

Supplementary Fig. 24 | Visual graphical recording of LSG neural activity after light inhibition. Representative LSG neural activity recordings of Beagles treatment with PtNP-shell or control at different time points.

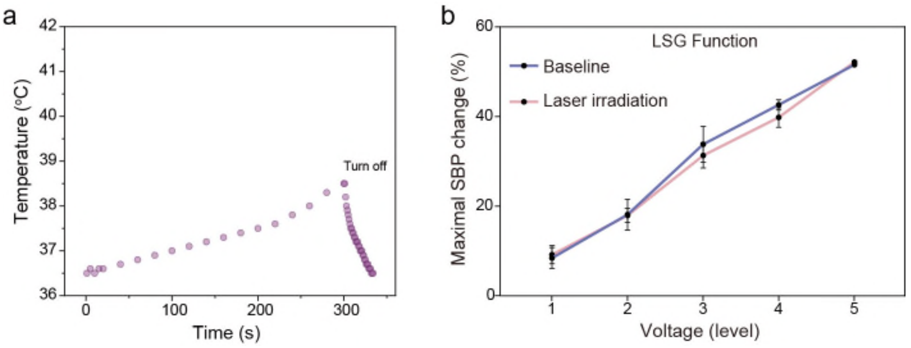

Supplementary Fig. 25 | Direct effect of NIR irradiation of LSG. a, Local temperature curve of LSG under NIR-II irradiation. **b**, Neural function of LSG before and after NIR-II irradiation. Source data are provided as a Source Data file.

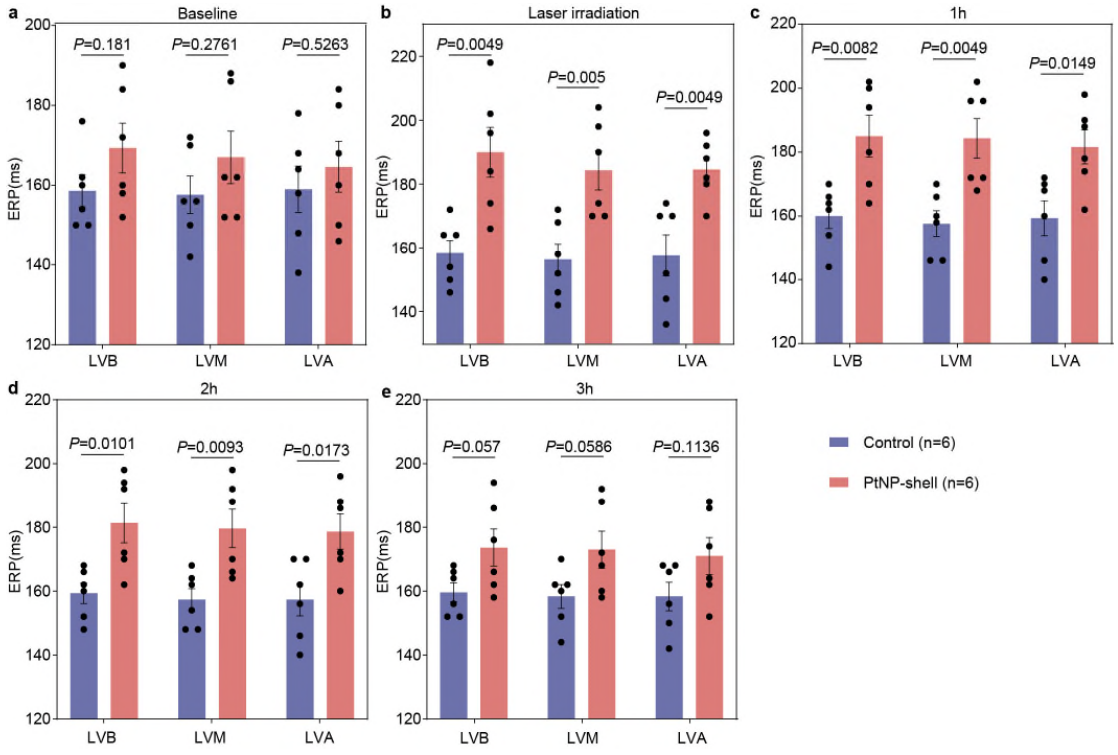

Supplementary Fig. 26 | Effects of PtNP-shell photothermal inhibition of LSG on ventricular electrophysiology. Effects on ventricular ERP at different sites in beagles treatment with PtNP-shell or control at different time points, n = 6 biologically independent replicates. Data are shown as the mean ± S.E.M. Unpaired two-tailed Student's t-test was applied for statistical analysis of (a–e). Source data are provided as a Source Data file.

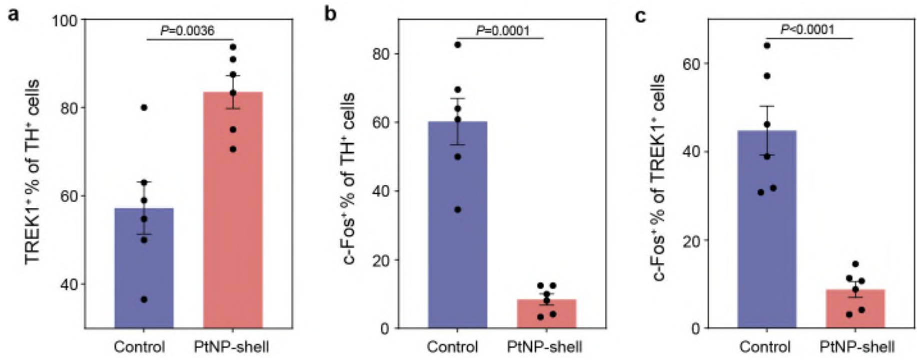

Supplementary Fig. 27 | Statistical analysis for the density of cells after photothermal inhibition of LSG tissues in different groups. a, Percentage of TREK1$^+$ cells within the TH$^+$ cell population. **b**, Percentage of c-Fos$^+$ cells within the TH$^+$ cell population. **c**, Percentage of c-Fos$^+$ cells within the TREK1$^+$ cell population, n = 6 biologically independent replicates. Data are shown as the mean ± S.E.M. Unpaired two-tailed Student's t-test was applied for statistical analysis of (a-c). Source data are provided as a Source Data file.

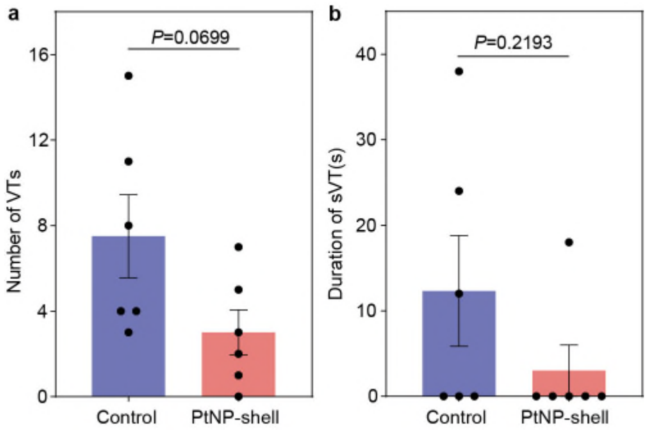

Supplementary Fig. 28 | Statistical analysis of recorded ventricular arrhythmia events posts myocardial ischemia. Quantitative analysis of the number of **a**, VTs and **b**, the duration of sVT of beagles with MI, n = 6 biologically independent replicates. Data are shown as the mean ± S.E.M. Unpaired two-tailed Student's t-test was applied for statistical analysis of (a,b). Source data are provided as a Source Data file.

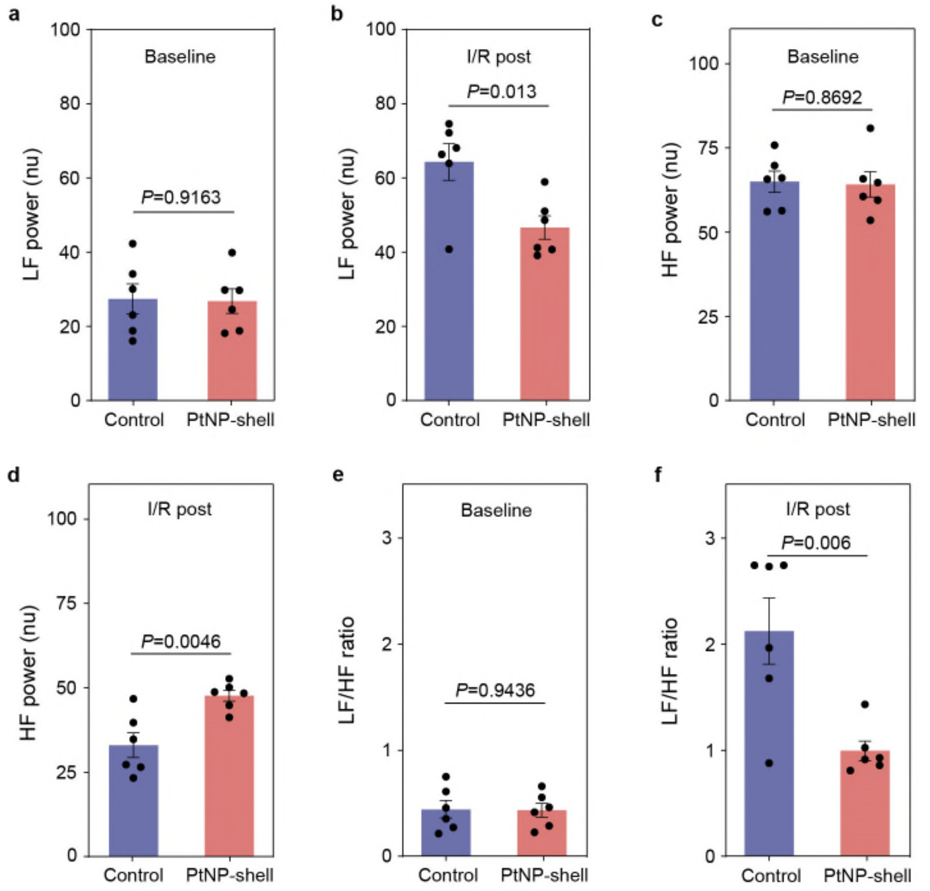

Supplementary Fig. 29 | Effect of photothermal inhibition of LSG on heart rate variability. Comparison of LF between the two groups at **a**, baseline and **b**, post myocardial I/R injury. Comparison of HF between the two groups at **c**, baseline and **d**, post myocardial I/R injury. Comparison of the ratio of LF/HF between the two groups at **e**, baseline and **f**, post myocardial I/R injury, n = 6 biologically independent replicates. Data are shown as the mean ± S.E.M. Unpaired two-tailed Student's t-test was applied for statistical analysis of (a-f). Source data are provided as a Source Data file.

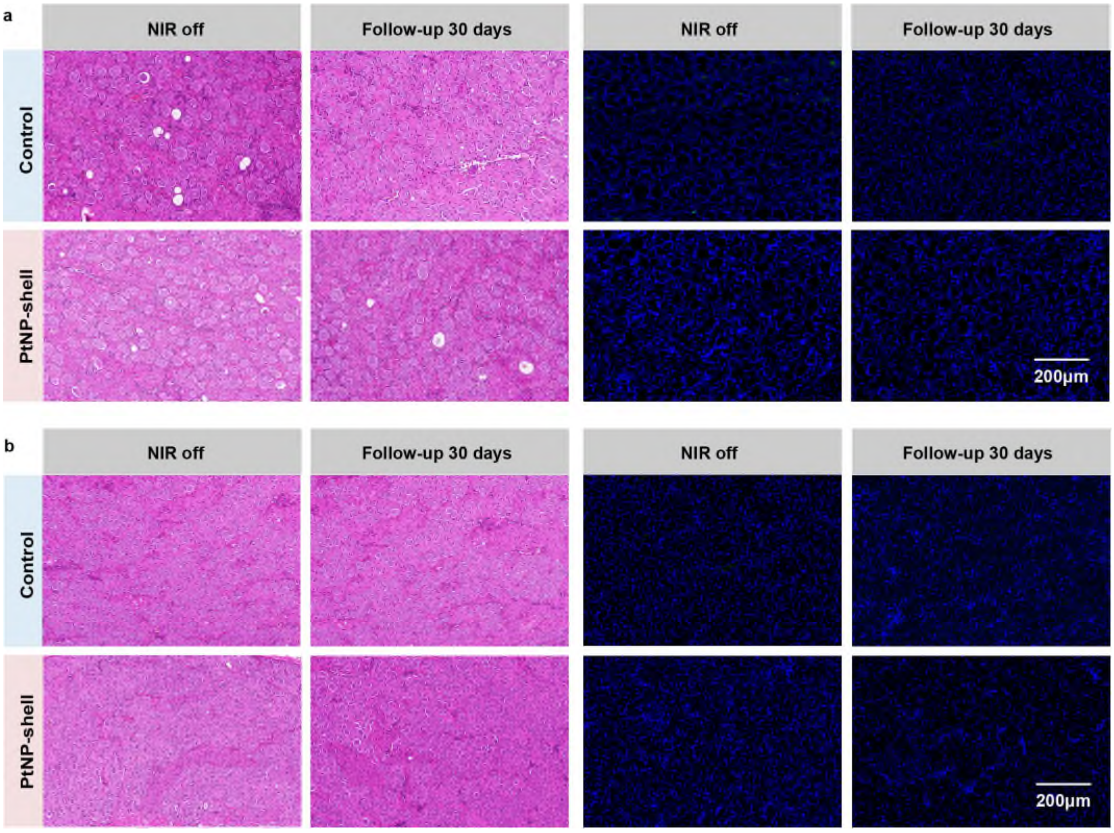

Supplementary Fig. 30 | Ganglion biocompatibility of targeted injections of PtNP or PBS after NIR-II irradiation and after 30 days of follow-up. a, Representative images of H&E and TUNEL staining of NG from different treatment groups immediately after NIR-II irradiation or after 30 days of follow-up. **b**, Representative images of H&E and TUNEL staining of LSG from different treatment groups immediately after NIR-II irradiation or after 30 days of follow-up.

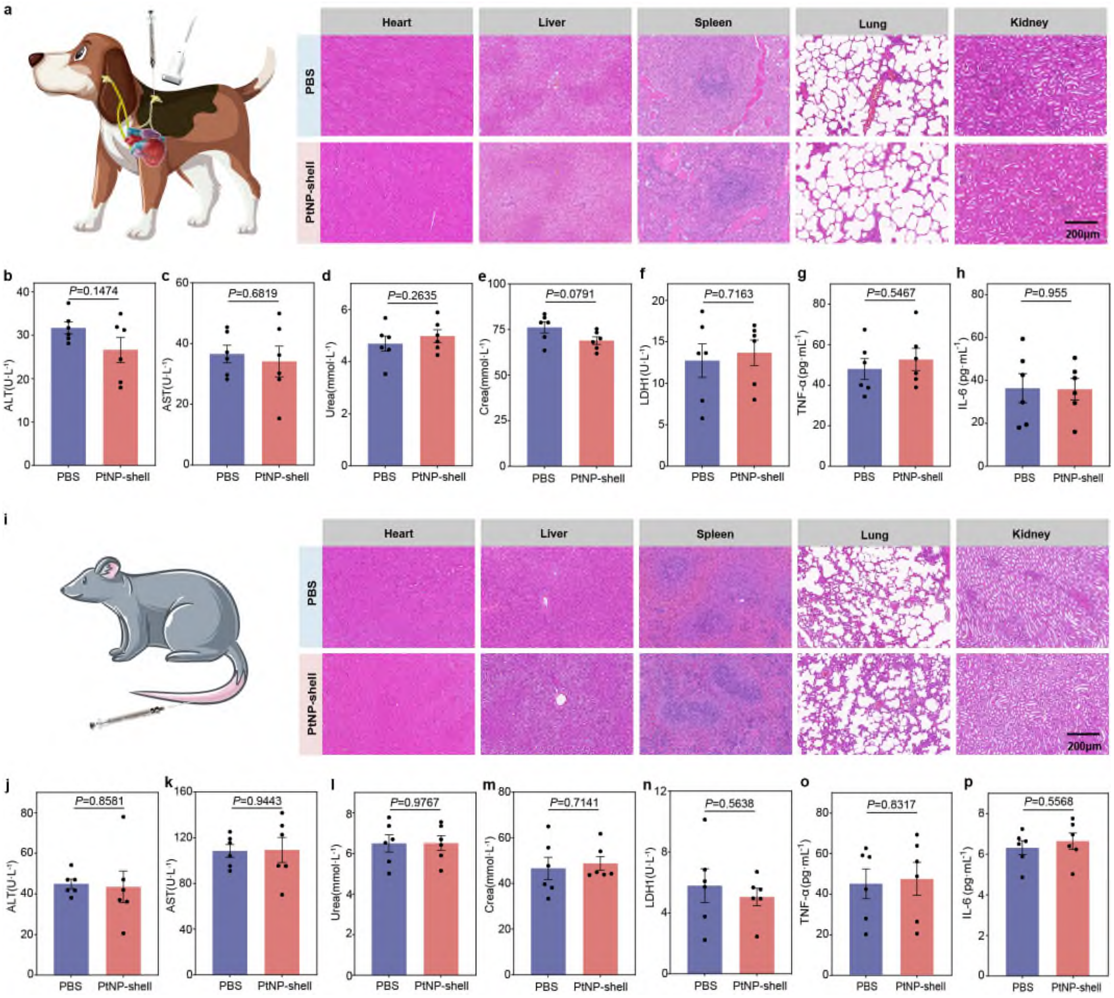

Supplementary Fig. 31 | Long term biosafety of PtNP-shell microinjection. Long-term in vivo biosafety was assessed by local injection of PtNP-shell into the ganglion of Beagle or by injection of equal doses of PtNP-shell into the tail vein of Sprague-Dawley rats. **a**, Representative H&E staining of major organs of beagles following different treatments. Blood biochemical analyses including **b**, ALT, **c**, AST, **d**, Urea, **e**, Crea, **f**, LDH1, **g**, TNF-α, and **h**, IL-6 were performed on Beagles in different treatment groups, n = 6 biologically independent replicates. **i**, Representative H&E staining of major organs of rats following different treatments. Blood biochemical analyses including **j**, ALT, **k**, AST, **l**, Urea, **m**, Crea, **n**, LDH1, **o**, TNF-α and **p**, IL-6 were performed on rats in different treatment groups, n = 6 biologically independent replicates. Unpaired two-tailed Student's t-test was applied for statistical analysis of (b-h, j-p). Source data are provided as a Source Data file.

Supplementary Table 1 | ξ potential of bare PtNP-shell, PtNP-shell + KOH and PtNP-shell@PEG. Source data are provided as a Source Data file.

	Zeta Potential (mV)	St. Dev. (mV)
PtNP-shell	45.8	5.8
PtNP-shell + KOH	–25.7	4.64
PtNP-shell@PEG	–19.9	3.69

Supplementary Table 2 | Comparison of photothermal conversion efficiency. Source data are provided as a Source Data file.

	Photothermal Conversion Efficiency (%)
This work	73.70
PEDOT:ICG@PEG-GTA[1]	71.10
MINDS[2]	71.00
PTG NPs[3]	67.60
RBC@Cu_{2-x}SeNPs[4]	67.20
AuDAg_2S[5]	67.10
MAPSULES[6]	67.00
Fe_3O_4@PPy@GOD NCs[7]	66.40
NPPBTPBF-BT[8]	66.40
AS1064[9]	65.92
Gold Nanoraspberry[10]	65.00
P-Pc-HSA[11]	64.70
Ultrathin polypyrrole nanosheets[12]	64.60
H_xMoO_3[13]	60.90
NiP PHNPs[14]	56.80
FP NRs[15]	56.60
COF[16]	55.20
2MPT$^{\cdot+}$-CB[17]	54.60
SPN-PT[18]	53.00
Pdots[19]	53.00
Pt Spirals[20]	52.50
TBDOPV-DT[21]	50.50
Ti_2O_3@HA NPs[22]	50.20
TBDOPV–DT NP[23]	50.00
DPP-IID-FA NPs[24]	49.50
SPN-DT[18]	49.00
NP[25]	49.00
FTQ nanoparticles[26]	49.00
CNPs[27]	49.00
H-SiO_x NPs[28]	48.60
BETA NPs[29]	47.60
CN-NPs[30]	47.60
Pt-NDs[31]	46.90
MoO_{3-x} nanobelts[32]	46.90
P3 NPs[33]	46.00
Ni_9S_8[34]	46.00
PtAg nanosheets[35]	45.70
Nb_2C NSs[36]	45.65
V_2C-TAT@Ex-RGD[37]	45.10
PEG-TONW NRs[38]	43.60
SPNI-II[39]	43.40
1T-MoS_2[40]	43.30
Bi@C NPs[41]	43.20
Au NPL@TiO_2[42]	42.10
CT NPs[43]	42.00
PPy-PEG NPs[44]	41.97
AuPt@CuS NSs[45]	41.56
$Bi_{19}S_{27}I_3$ nanorods[46]	41.50
Cu_3BiS_3 NR[47]	40.70
MPAE-NPS[48]	40.07

Supplementary references

1 Li, L. Y. *et al.* PEDOT nanocomposites mediated dual-modal photodynamic and photothermal targeted sterilization in both NIR I and II window. *Biomaterials* **41**, 132–140 (2015).

2 Wu, X. *et al.* Tether-free photothermal deep-brain stimulation in freely behaving mice via wide-field illumination in the near-infrared-II window. *Nat. Biomed. Eng.* **6**, 754–770 (2022).

3 Chen, J. *et al.* Atomically precise photothermal nanomachines. *Nat. Mater.* **23**, 271–280 (2024).

4 Liu, Z. *et al.* Fabrication of red blood cell membrane-camouflaged $Cu_{2-x}Se$ nanoparticles for phototherapy in the second near-infrared window. *Chem. Commun.* **55**, 6523–6526 (2019).

5 He, J. *et al.* SERS/NIR-II optical nanoprobes for multidimensional tumor imaging from living subjects, pathology, and single cells and guided NIR-II photothermal therapy. *Adv. Funct. Mater.* **32**, 2208028 (2022).

6 Fluksman, A. *et al.* Efficient tumor eradication at ultralow drug concentration via externally controlled and boosted metallic iron magnetoplasmonic nanocapsules. *ACS Nano* **17**, 1946–1958 (2022).

7 Feng, W. *et al.* Nanocatalysts-augmented and photothermal-enhanced tumor-specific sequential nanocatalytic therapy in both NIR-I and NIR-II biowindows. *Adv. Mater.* **31**, 1805919 (2019).

8 Cao, Z. *et al.* Semiconducting polymer-based nanoparticles with strong absorbance in NIR-II window for in vivo photothermal therapy and photoacoustic imaging. *Biomaterials* **155**, 103–111 (2018).

9 Dai, Y. *et al.* Precise tailoring of mesoporous silica-coated gold nanorods for laser ignition at 1064 nm. *ACS Appl. Nano Mater.* **6**, 4946–4956 (2023).

10 Sangnier, A. P. *et al.* Raspberry-like small multicore gold nanostructures for efficient photothermal conversion in the first and second near-infrared windows. *Chem. Commun.* **55**, 4055–4058 (2019).

11 Zhou, L. N. *et al.* Single-molecular phosphorus phthalocyanine-based near-infrared-II nanoagent for photothermal antitumor therapy. *RSC Adv.* **10**, 22656–22662 (2020).

12 Wang, X., Ma, Y. C., Sheng, X., Wang, Y. C. & Xu, H. X. Ultrathin polypyrrole nanosheets via space-confined synthesis for efficient photothermal therapy in the second near-Infrared window. *Nano Lett.* **18**, 2217–2225 (2018).

13 Zhu, Q. *et al.* Hydrogenated oxide material for self-targeting and automatic-degrading photothermal tumor therapy in the NIR-II bio-window. *Adv. Funct. Mater.* **32**, 2110881 (2021).

14 Liu, Y. *et al.* Double switch biodegradable porous hollow trinickel monophosphide nanospheres for multimodal imaging guided photothermal therapy. *Nano Lett.* **19**, 5093–5101 (2019).

15 Liu, Y. *et al.* One-dimensional Fe_2P acts as a fenton agent in response to NIR II light and ultrasound for deep tumor synergetic theranostics. *Angew. Chem. Int. Ed.* **58**, 2407–2412 (2019).

16 Mi, Z. *et al.* Stable radical cation-containing covalent organic frameworks exhibiting remarkable structure-enhanced photothermal conversion. *J. Am. Chem. Soc.* **141**, 14433–14442

(2019).

17 Tang, B. H. *et al.* A supramolecular radical dimer: high-efficiency NIR-II photothermal conversion and therapy. *Angew. Chem. Int. Ed.* **58**, 15526–15531 (2019).

18 Jiang, Y. Y. *et al.* Metabolizable semiconducting polymer nanoparticles for second near-Infrared photoacoustic imaging. *Adv. Mater.* **31**, 1808166 (2019).

19 Men, X. *et al.* Ultrasmall semiconducting polymer dots with rapid clearance for second near-infrared photoacoustic imaging and photothermal cancer therapy. *Adv. Funct. Mater.* **30**, 1909673 (2020).

20 Wang, Q. S. *et al.* Plasmonic Pt superstructures with boosted near-infrared absorption and photothermal conversion efficiency in the second biowindow for cancer therapy. *Adv. Mater.* **31**, 1904836 (2019).

21 Cao, Y. Y. *et al.* Highly efficient NIR-II photothermal conversion based on an organic conjugated polymer. *Chem. Mater.* **29**, 718–725 (2017).

22 Zeng, J. *et al.* Facile preparation of biocompatible Ti2O3 nanoparticles for second near-infrared window photothermal therapy. *J. Mater. Chem. B* **6**, 7889–7897 (2018).

23 Sun, T. T. *et al.* Second near-infrared conjugated polymer nanoparticles for photoacoustic imaging and photothermal therapy. *ACS Appl. Mater. Interfaces* **10**, 7919–7926 (2018).

24 Wei, Z. W. *et al.* Semiconducting polymer-based nanoparticles for photothermal therapy at the second near-infrared window. *Chem. Commun.* **54**, 13599–13602 (2018).

25 Yu, Y. *et al.* Biodegradable polymer with effective near-infrared-II absorption as a photothermal agent for deep tumor therapy. *Adv. Mater.* **34**, 2105976 (2021).

26 Wu, Z. H. *et al.* A terrylene–anthraquinone dyad as a chromophore for photothermal therapy in the NIR-II window. *J. Am. Chem. Soc.* **145**, 26487–26493 (2023).

27 Jia, W. Y. *et al.* Novel conjugated small molecule-based nanoparticles for NIR-II photothermal antibacterial therapy. *Chem. Commun.* **58**, 6340–6343 (2022).

28 Yu, X. J., Yang, K., Chen, X. Y. & Li, W. W. Black hollow silicon oxide nanoparticles as highly efficient photothermal agents in the second near-infrared window for in vivo cancer therapy. *Biomaterials* **143**, 120–129 (2017).

29 Chen, S. *et al.* Increasing molecular planarity through donor/side-chain engineering for improved NIR-IIa fluorescence imaging and NIR-II photothermal therapy under 1064 nm. *Angew. Chem. Int. Ed.* **62**, 202215372 (2022).

30 Tian, Y. *et al.* Extended π-conjugative carbon nitride for single 1064 nm laser-activated photodynamic/photothermal synergistic therapy and photoacoustic imaging. *ACS Appl. Mater. Interfaces* **14**, 7626–7635 (2022).

31 Tang, Y. A. *et al.* Albumin-coordinated assembly of clearable platinum nanodots for photo-induced cancer theranostics. *Biomaterials* **154**, 248–260 (2018).

32 Zhou, Z. *et al.* Activating layered metal oxide nanomaterials via structural engineering as biodegradable nanoagents for photothermal cancer therapy. *Small* **17**, 2007486 (2021).

33 Sun, T. T. *et al.* Tailor-made semiconducting polymers for second near-infrared photothermal therapy of orthotopic liver cancer. *ACS Nano* **13**, 7345–7354 (2019).

34 Lei, Z. Y. *et al.* A full-spectrum-absorption from nickel sulphide nanoparticles for efficient

NIR-II window photothermal therapy. *Nanoscale* **11**, 20161–20170 (2019).

35 Zhang, Y. *et al.* Ultrathin two-dimensional plasmonic PtAg nanosheets for broadband phototheranostics in both NIR-I and NIR-II biowindows. *Adv. Sci.* **8**, 2100386 (2021).

36 Lin, H., Gao, S. S., Dai, C., Chen, Y. & Shi, J. L. A two-dimensional biodegradable niobium carbide (MXene) for photothermal tumor eradication in NIR-I and NIR-II biowindows. *J. Am. Chem. Soc.* **139**, 16235–16247 (2017).

37 Cao, Y. *et al.* Engineered exosome-mediated near-infrared-II region V_2C quantum dot delivery for nucleus-target low-temperature photothermal therapy. *ACS Nano* **13**, 1499–1510 (2019).

38 Cheng, Y. R. *et al.* Ultrathin tellurium oxide/ammonium tungsten bronze nanoribbon for multimodality imaging and second near-infrared region photothermal therapy. *Nano Lett.* **19**, 1179–1189 (2019).

39 Jiang, Y. Y., Li, J. C., Zhen, X., Xie, C. & Pu, K. Y. Dual-peak absorbing semiconducting copolymer nanoparticles for first and second near-infrared window photothermal therapy: a comparative study. *Adv. Mater.* **30**, 1705980 (2018).

40 Zhou, Z. *et al.* Metallic 1T phase enabling MoS_2 nanodots as an efficient agent for photoacoustic imaging guided photothermal therapy in the near-Infrared-II window. *Small* **16**, 2004173 (2020).

41 Zhen, W. Y. *et al.* Gram-scale fabrication of Bi@C nanoparticles through one-step hydrothermal method for dual-model imaging-guided NIR-II photothermal therapy. *Nanoscale* **11**, 9906–9911 (2019).

42 Gao, F. L. *et al.* Titania-coated 2D gold nanoplates as nanoagents for synergistic photothermal/sonodynamic therapy in the second near-infrared window. *Nanoscale* **11**, 2374–2384 (2019).

43 Tian, S. *et al.* Water-soluble organic nanoparticles with programable intermolecular charge transfer for NIR-II photothermal anti-bacterial therapy. *Angew. Chem. Int. Ed.* **60**, 11758–11762 (2021).

44 Zeng, W. W. *et al.* Renal-clearable ultrasmall polypyrrole nanoparticles with size-regulated property for second near-infrared light-mediated photothermal therapy. *Adv. Funct. Mater.* **31**, 2008362 (2021).

45 Cai, R. *et al.* Plasmonic AuPt@CuS heterostructure with enhanced synergistic efficacy for radiophotothermal therapy. *J. Am. Chem. Soc.* **143**, 16113–16127 (2021).

46 Xiong, J. *et al.* $Bi_{19}S_{27}I_3$ nanorods: a new candidate for photothermal therapy in the first and second biological near-infrared windows. *Nanoscale* **13**, 5369–5382 (2021).

47 Li, A. *et al.* Synergistic thermoradiotherapy based on PEGylated Cu_3BiS_3 ternary semiconductor nanorods with strong absorption in the second near-infrared window. *Biomaterials* **112**, 164–175 (2017).

48 Cheng, Q. *et al.* Antiquenching macromolecular NIR-II probes with high-contrast brightness for imaging-guided photothermal therapy under 1064 nm irradiation. *Adv. Healthc. Mater.* **11**, 2101697 (2021).
