# Supplementary material for: Pt nanoshells with a high NIR-II photothermal conversion efficiency mediates multimodal neuromodulation against ventricular arrhythmias
Source: Nat Commun. 2024 Jul 28;15:6362. doi: 10.1038/s41467-024-50557-w (PMC11284209; doi:10.1038/s41467-024-50557-w)
Supplement: Supplementary file 2 — Reporting Summary [file 41467_2024_50557_MOESM2_ESM.pdf]

nature portfolio

Corresponding author(s): Xiaoya Zhou, Lilei Yu, Lei Fu

Last updated by author(s): Jun 28, 2024

Reporting Summary

Nature Portfolio wishes to improve the reproducibility of the work that we publish. This form provides structure for consistency and transparency in reporting. For further information on Nature Portfolio policies, see our Editorial Policies and the Editorial Policy Checklist.

Statistics

For all statistical analyses, confirm that the following items are present in the figure legend, table legend, main text, or Methods section.

n/a	Confirmed	
☐	☒	The exact sample size (*n*) for each experimental group/condition, given as a discrete number and unit of measurement
☐	☒	A statement on whether measurements were taken from distinct samples or whether the same sample was measured repeatedly
☐	☒	The statistical test(s) used AND whether they are one- or two-sided<br>*Only common tests should be described solely by name; describe more complex techniques in the Methods section.*
☐	☒	A description of all covariates tested
☐	☒	A description of any assumptions or corrections, such as tests of normality and adjustment for multiple comparisons
☐	☒	A full description of the statistical parameters including central tendency (e.g. means) or other basic estimates (e.g. regression coefficient) AND variation (e.g. standard deviation) or associated estimates of uncertainty (e.g. confidence intervals)
☐	☒	For null hypothesis testing, the test statistic (e.g. *F*, *t*, *r*) with confidence intervals, effect sizes, degrees of freedom and *P* value noted<br>*Give P values as exact values whenever suitable.*
☒	☐	For Bayesian analysis, information on the choice of priors and Markov chain Monte Carlo settings
☐	☒	For hierarchical and complex designs, identification of the appropriate level for tests and full reporting of outcomes
☐	☒	Estimates of effect sizes (e.g. Cohen's *d*, Pearson's *r*), indicating how they were calculated

Our web collection on statistics for biologists contains articles on many of the points above.

Software and code

Policy information about availability of computer code

Data collection	Lead 7000 Computerized Laboratory System (Jinjiang, Chengdu, China); Power Lab data acquisition system (ADInstruments, New South Wales, Australia)
Data analysis	Labchart software (version 8.0, ADInstruments); GraphPad Prism 9.0 software (GraphPad software, Inc., La Jolla, CA, USA); Oringinpro 2018 (OriginLab, USA)

For manuscripts utilizing custom algorithms or software that are central to the research but not yet described in published literature, software must be made available to editors and reviewers. We strongly encourage code deposition in a community repository (e.g. GitHub). See the Nature Portfolio guidelines for submitting code & software for further information.

Data

Policy information about availability of data

All manuscripts must include a data availability statement. This statement should provide the following information, where applicable:

- Accession codes, unique identifiers, or web links for publicly available datasets
- A description of any restrictions on data availability
- For clinical datasets or third party data, please ensure that the statement adheres to our policy

Source data are provided with this paper.

Research involving human participants, their data, or biological material

Policy information about studies with human participants or human data. See also policy information about sex, gender (identity/presentation), and sexual orientation and race, ethnicity and racism.

Reporting on sex and gender	*Use the terms sex (biological attribute) and gender (shaped by social and cultural circumstances) carefully in order to avoid confusing both terms. Indicate if findings apply to only one sex or gender; describe whether sex and gender were considered in study design; whether sex and/or gender was determined based on self-reporting or assigned and methods used. Provide in the source data disaggregated sex and gender data, where this information has been collected, and if consent has been obtained for sharing of individual-level data; provide overall numbers in this Reporting Summary. Please state if this information has not been collected. Report sex- and gender-based analyses where performed, justify reasons for lack of sex- and gender-based analysis.*
Reporting on race, ethnicity, or other socially relevant groupings	*Please specify the socially constructed or socially relevant categorization variable(s) used in your manuscript and explain why they were used. Please note that such variables should not be used as proxies for other socially constructed/relevant variables (for example, race or ethnicity should not be used as a proxy for socioeconomic status). Provide clear definitions of the relevant terms used, how they were provided (by the participants/respondents, the researchers, or third parties), and the method(s) used to classify people into the different categories (e.g. self-report, census or administrative data, social media data, etc.) Please provide details about how you controlled for confounding variables in your analyses.*
Population characteristics	*Describe the covariate-relevant population characteristics of the human research participants (e.g. age, genotypic information, past and current diagnosis and treatment categories). If you filled out the behavioural & social sciences study design questions and have nothing to add here, write "See above."*
Recruitment	*Describe how participants were recruited. Outline any potential self-selection bias or other biases that may be present and how these are likely to impact results.*
Ethics oversight	*Identify the organization(s) that approved the study protocol.*

Note that full information on the approval of the study protocol must also be provided in the manuscript.

Field-specific reporting

Please select the one below that is the best fit for your research. If you are not sure, read the appropriate sections before making your selection.

☒ Life sciences ☐ Behavioural & social sciences ☐ Ecological, evolutionary & environmental sciences

For a reference copy of the document with all sections, see nature.com/documents/nr-reporting-summary-flat.pdf

Life sciences study design

All studies must disclose on these points even when the disclosure is negative.

Sample size	36 Beagles and 12 rats were used for the experiments, including 12 dogs for the acute MI model, 12 dogs for the acute I/R model, and 12 dogs and 12 rats for the safety experiments
Data exclusions	Data were analyzed after they were obtained from the experiment, and no data were artificially excluded.
Replication	All of our cell experiments were performed for more than 3 repetitions, and animal experiments were performed for more than 6 repetitions to ensure the reproducibility and stability of the results.
Randomization	The experimental animals were randomly grouped
Blinding	This study is single-blind, and the researchers who designed the experiment are aware of the grouping situation. However, the experimenters who perform modeling, data measurement, recording and analysis are not clear about grouping.

Reporting for specific materials, systems and methods

We require information from authors about some types of materials, experimental systems and methods used in many studies. Here, indicate whether each material, system or method listed is relevant to your study. If you are not sure if a list item applies to your research, read the appropriate section before selecting a response.

Materials & experimental systems

n/a	Involved in the study	
☐	☒	Antibodies
☐	☒	Eukaryotic cell lines
☒	☐	Palaeontology and archaeology
☐	☒	Animals and other organisms
☒	☐	Clinical data
☒	☐	Dual use research of concern
☒	☐	Plants

Methods

n/a	Involved in the study	
☒	☐	ChIP-seq
☒	☐	Flow cytometry
☒	☐	MRI-based neuroimaging

Antibodies

Antibodies used

Anti-TRPV1, anti-c-Fos, anti-VAChT, anti-TH, anti-TREK1

Validation

Rabbit monoclonal anti-TRPV1 antibody (Cat. No. A23386, Clone No. ARC57842, lot No. 6100001378) used for protein blotting were purchased from ABclonal (Wuhan, China). Mouse monoclonal anti-TREK1 antibody (Cat. No. sc-398449, Clone No. F-6, lot No. 13022) used in western blot and immunofluorescence staining was purchased from Santa Cruz Biotechnology (Texas, U.S.). Rabbit monoclonal Anti-c-Fos (Cat. No. A24620, Clone No. ARC63309, lot No.6100003655), rabbit polyclonal anti-VAChT (Cat. No. A16068, lot No. 3521739003), rabbit polyclonal anti-TH (Cat. No. A12756, lot No. 550036579) and rabbit polyclonal Anti-TRPV1 (Cat. No. A8564, lot No. 3523032113) antibodies used in immunofluorescence staining were purchased from ABclonal. Glyceraldehyde 3-phosphate dehydrogenase (GAPDH) was purchased from Servicebio (Wuhan, China).

Eukaryotic cell lines

Policy information about cell lines and Sex and Gender in Research

Cell line source(s)

These cell lines (HT-22; HEK293T)were purchased from Procell (Wuhan, China).

Authentication

All cell lines have been authenticated using STR profiling.HT22 and HEK293T cells were validated for STR at Biowing (Shanghai, China).

Mycoplasma contamination

All cell lines screened for mycoplasma contamination and ensure all cell lines uesd are free of mycoplasma contamination.

Commonly misidentified lines (See ICLAC register)

We did not use any misidentified cell lines

Animals and other research organisms

Policy information about studies involving animals; ARRIVE guidelines recommended for reporting animal research, and Sex and Gender in Research

Laboratory animals

The experimental animals included dogs, rats, and cells. Adult male Beagles aged around 12 months and weighing 10-12 kg. Male Sprague-Dawley rats aged 6-8 weeks, weighing 220-250g

Wild animals

Purchase specialized laboratory animals rather than wild animals

Reporting on sex

The experimental beagles we utilized were exclusively male, aligning with the conventional cardiac electrophysiology research on animal sex (Adv. Mater. 2023, 2304620; Circ.-Heart Fail. 2014, 7, 1014–1021). The selection was aimed at controlling for variables that could potentially impact the experiment's outcomes. It is widely acknowledged that estrogen exerts a protective effect on nerves and the heart, possibly even inhibiting inflammation (Neural Regen. Res. 2022, 17, 1629–1632). Moreover, the menstrual cycle exerts an influence on the expression of hormones and nerve activity in female experimental animals. (Nat. Mental Health 2023, 1, 761–771). Considering the physiological characteristics of females, we therefore chose male beagles as the experimental subjects in this study. We have indicated the sex of the experimental beagles in the methods.

Field-collected samples

The animal experimental operations are located in a standard surgical operation laboratory. The parameters of light and temperature were controlled and did not influence the conditions of our intervention. At the end of the acute experiment, the anesthetized animals were terminated by measuring the ventricular fibrillation threshold and collecting the needed samples.

Ethics oversight

All animal experiments were approved by the Animal Care and Use Committee of Renmin Hospital of Wuhan University (WDRM20230805A).

Note that full information on the approval of the study protocol must also be provided in the manuscript.

Plants

Seed stocks

Report on the source of all seed stocks or other plant material used. If applicable, state the seed stock centre and catalogue number. If plant specimens were collected from the field, describe the collection location, date and sampling procedures.

Novel plant genotypes

Describe the methods by which all novel plant genotypes were produced. This includes those generated by transgenic approaches, gene editing, chemical/radiation-based mutagenesis and hybridization. For transgenic lines, describe the transformation method, the number of independent lines analyzed and the generation upon which experiments were performed. For gene-edited lines, describe the editor used, the endogenous sequence targeted for editing, the targeting guide RNA sequence (if applicable) and how the editor was applied.

Authentication

Describe any authentication procedures for each seed stock used or novel genotype generated. Describe any experiments used to assess the effect of a mutation and, where applicable, how potential secondary effects (e.g. second site T-DNA insertions, mosiacism, off-target gene editing) were examined.
